# Detection of a combination of serum IgG and IgA antibodies against selected mycobacterial targets provides promising diagnostic signatures for active TB

**DOI:** 10.18632/oncotarget.16401

**Published:** 2017-03-21

**Authors:** Dolapo O. Awoniyi, Ralf Baumann, Novel N. Chegou, Belinda Kriel, Ruschca Jacobs, Martin Kidd, Andre G. Loxton, Susanne Kaempfer, Mahavir Singh, Gerhard Walzl

**Affiliations:** ^1^ DST/NRF Centre of Excellence for Biomedical TB Research and SAMRC Centre for TB Research, Division of Molecular Biology and Human Genetics, Department of Biomedical Sciences, Faculty of Medicine and Health Sciences, Stellenbosch University, Cape Town, South Africa; ^2^ Institute for Occupational and Social Medicine, Aachen University of Technology, Aachen, Germany; ^3^ Lionex Diagnostics and Therapeutics, Braunschweig, Germany; ^4^ Centre for Statistical Analysis, Stellenbosch University, Stellenbosch, South Africa

**Keywords:** tuberculosis, diagnosis, biomarker, antibody, Ig class

## Abstract

Immunoglobulin G (IgG) based tests for the diagnosis of active tuberculosis (TB) disease often show a lack of specificity in TB endemic regions, which is mainly due to a high background prevalence of LTBI. Here, we investigated the combined performance of the responses of different Ig classes to selected mycobacterial antigens in primary healthcare clinic attendees with signs and symptoms suggestive of TB. The sensitivity and specificity of IgA, IgG and/or IgM to LAM and 7 mycobacterial protein antigens (ESAT-6, Tpx, PstS1, AlaDH, MPT64, 16kDa and 19kDa) and 2 antigen combinations (TUB, TB-LTBI) in the plasma of 63 individuals who underwent diagnostic work-up for TB after presenting with symptoms and signs compatible with possible active TB were evaluated. Active TB was excluded in 42 individuals of whom 21 has LTBI whereas active TB was confirmed in 21 patients of whom 19 had a follow-up blood draw at the end of 6-month anti-TB treatment. The leading single serodiagnostic markers to differentiate between the presence or absence of active TB were anti-16 kDa IgA, anti-MPT64 IgA with sensitivity and specificity of 90%/90% and 95%/90%, respectively. The combined use of 3 or 4 antibodies further improved this performance to accuracies above 95%. After successful completion of anti-TB treatment at month 6, the levels of 16 kDa IgA and 16 kDa IgM dropped significantly whereas LAM IgG and TB-LTBI IgG increased. These results show the potential of extending investigation of anti-tuberculous IgG responses to include IgM and IgA responses against selected protein and non-protein antigens in differentiating active TB from other respiratory diseases in TB endemic settings.

## INTRODUCTION

Tuberculosis (TB) still remains a global threat to mankind and although the millennium development goals target of halting and reversing the increasing incidence of TB globally was achieved, TB still killed 1.5 million people in 2014 [1]. The currently available diagnostic tools have many limitations including poor sensitivity (smear microscopy), long turn-around time (culture), the use of expensive tools and the difficulty to develop these tests into point-of-care (POC) tests [2]. The high prevalence of latent tuberculosis infection (LTBI), in addition to high TB and HIV co-infection in resource-poor settings such as in Africa, calls for the development of rapid diagnostic tools, especially ones that discriminate between active TB and LTBI. The use of commercial serological tests for diagnosing active TB has been strongly criticised, as a result of the poor accuracy of commercial tests in TB endemic settings [3], which is largely due to a high prevalence of LTBI [4–6]. However, further research in the field of antibody-based tests has been encouraged, as serological tests lend itself to the development of POC tests. Furthermore, it needs to be ascertained to what degree the lack of serological test specificity is due to a subgroup of LTBI with high risk for progression to active TB [4].

The standard strategy for TB treatment, directly observed treatment short course (DOTS) consists of a two month period of four drugs followed by another four months of two anti-TB drugs [7]. The necessity of a treatment period of six month regimen is largely due to persistent bacilli that are not rapidly killed [8] and these persister organisms can be the cause of treatment failure and relapse [9]. The identification of better surrogate markers of treatment response than sputum culture would be a major boost towards enhancing treatment monitoring [10] and the potential role of serologic tests needs to be evaluated [11].

The main purpose of the present study was to evaluate the potential of IgG, IgA and IgM serodiagnostic markers for the diagnosis of active TB disease among people presenting with presumed TB at primary health care clinics and to explore their potential as treatment response markers.

## RESULTS

### Clinical and demographic characteristics of study participants

A total of 63 participants were included in the study. Out of these 63 individuals, 33 (52%) were females. The mean age of all study participants was 34.1±11.3 years and only one of the study participants was HIV positive. Using a pre-established diagnostic algorithm [12], 21 patients were classified as having TB disease. Of the 42 patients with other respiratory diseases (ORD), 21 were QFT-negative (*M. tuberculosis* uninfected) while 21 individuals were latently infected (LTBI), as defined by a positive QFT-test using the manufacturer's recommended cut-off value (≥0.35 IU/ml). Table 1 shows the demographic and baseline characteristics of the study participants.

**Table 1:** Demographic characteristics of study participants

	All	TB	LTBI	QFT-negative ORD
Participants no.	63	21	21	21
Age, yr	34.1±11.3	41.0±10.5	26.0±3.6	35.2±12.2
M/F no. (%)	30(48)/33(52)	8(38)/13(62)	13(62)/8(38)	9(43)/12(57)
HIV status pos/neg	1/62	0/20	0/20	1/20
QFT-IT positive	42	21	21	Nil

Values are mean (±SD) unless indicated otherwise.

### Evaluation of antibodies for the diagnosis of active TB disease

**Figure 1:**
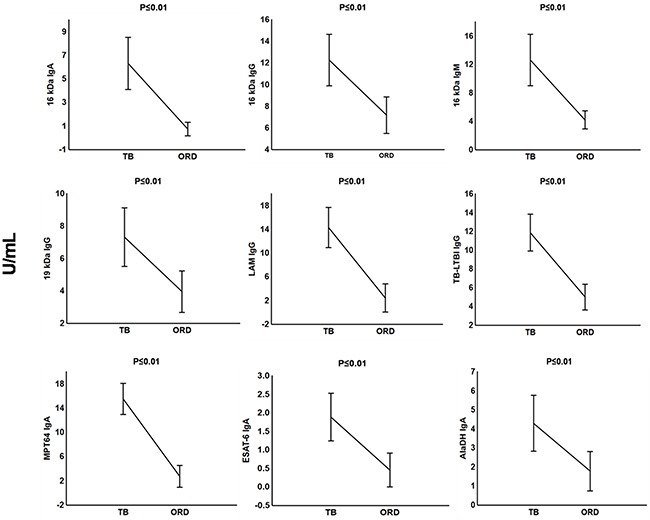
Plasma concentrations of serodiagnostic markers in TB and ORD Concentrations of plasma serodiagnostic markers were measured in 21 TB patients, and 42 ORD cases. The ORD group comprised 21 LTBI individuals and 21 QFT-negative ORD. Representative plots are shown for diagnostic markers showing significant differences between groups and the vertical bars denote the mean and 95% confidence intervals. Corrections were applied within analyses of markers using Fischer Least Significant Differences post hoc test or Games- Howell post hoc test depending on homogeneity of variance. All the reported significant p-values were adjusted.

**Figure 2:**
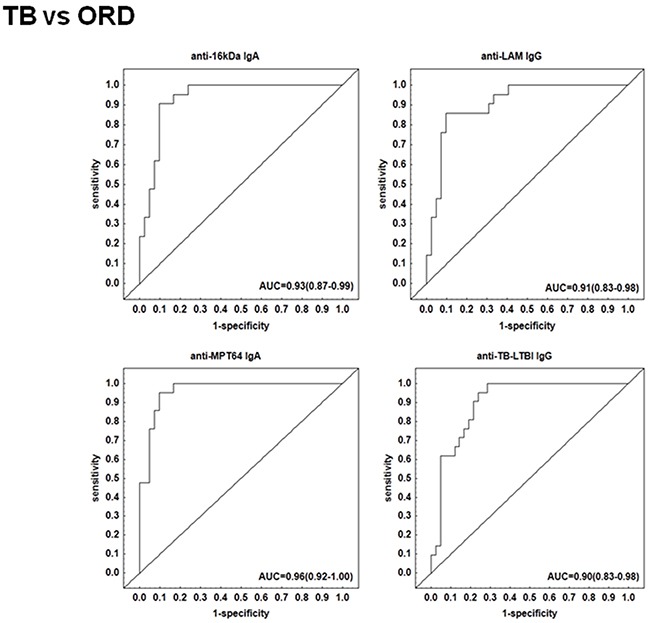
Receiver operator characteristics (ROC) curves of top single serodiagnostic markers for discriminating 21 active tuberculosis patients from 42 other respiratory disease cases

The preparation of the recombinant proteins of *M. tuberculosis* antigens and the pre-coated IgG, IgA and IgM ELISA test kits by LIONEX Diagnostics and Therapeutics, Braunschweig, Germany has been described in detail in the respective methods section. In South Africa, we then measured and analyzed the titres of IgG, IgA and/or IgM antibodies against lipoarabinomannan (LAM) and 7 mycobacterial protein antigens (ESAT-6, Tpx, PstS1, AlaDH, MPT64, 16kDa and 19kDa) and 2 antigen combinations (TUB, TB-LTBI) in plasma samples obtained from all 63 study participants (Table 1). When the antibody titres in the TB patients were compared to the titres obtained in all individuals with other respiratory diseases ORD (regardless of QFT results), IgA antibodies against 16 kDa, AlaDH, ESAT-6, MPT64, IgG antibodies against 16 kDa, 19 kDa, LAM, TB-LTBI and IgM antibodies against 16 kDa antigen were significantly higher in TB patients compared to non-cases (Figure 1). ROC curve analysis indicated that, anti-LAM IgG, anti-TB-LTBI IgG, anti-MPT64 IgA, and anti-16 kDa IgA antibodies were the leading single serodiagnostic markers (Table 2). Anti-MPT64 and 16 kDa antigen IgA ascertained TB disease with areas under the ROC curves (AUC) of 0.96 (95% CI, 0.92-1.00) and 0.93 (95% CI, 0.87-0.99), respectively. The corresponding sensitivities and specificities were 95% / 90% and 90% / 90%, respectively (Table 2 and Figure 2).

**Table 2:** Sensitivities and specificities of single serodiagnostic markers in differentiating active TB (n=21) from LTBI (n=21) and ORD (n=42)

Antigen	Ig class	AUC	TB vs ORD	AUC	TB vs LTBI
		(95% CI)	Sensitivity/specificity (%)	(95% CI)	Sensitivity/specificity (%)
AlaDH	A	0.73(0.59-0.86)	0.76/0.69	0.73(0.57-0.89)	0.76/0.71
AlaDH	G	0.63(0.49-0.76)	0.62/0.64	0.65(0.47-0.83)	0.76/0.57
ESAT-6	A	0.73(0.60-0.86)	0.67/0.79	0.75(0.61-0.89)	0.67/0.81
ESAT-6	G	0.46(0.27-0.65)	0.52/0.60	0.45(0.25-0.64)	0.52/0.52
LAM	A	0.64(0.49-0.79)	0.52-0.76	0.59(0.41-0.76)	0.52/0.76
LAM	G	0.91(0.83-0.98)	0.86/0.90	0.95(0.89-1.00)	0.86/0.95
MPT64	A	0.96(0.92-1.00)	0.95/0.90	0.97(0.91-1.00)	0.95/0.90
MPT64	G	0.69(0.56-0.82)	0.76/0.60	0.71(0.54-0.88)	0.76/0.67
PstS1	G	0.43(0.28-0.58)	0.52/0.52	0.51(0.32-0.70)	0.62/0.62
TB-LTBI	A	0.76(0.63-0.88)	0.67/0.76	0.76(0.61-0.91)	0.67/0.86
TB-LTBI	G	0.90(0.83-0.98)	0.90/0.79	0.89(0.78-0.99)	0.90/0.71
Tpx	G	0.54(0.39-0.68)	0.55/0.57	0.52(0.34-0.70)	0.57/0.52
TUB	A	0.74(0.62-0.87)	0.81/0.60	0.79(0.65-0.92)	0.81/0.62
16kDa	A	0.93(0.87-0.99)	0.90/0.90	0.99(0.98-1.00)	0.95/0.95
16kDa	G	0.77(0.66-0.89)	0.81/0.67	0.78(0.63-0.93)	0.95/0.62
16kDa	M	0.84(0.74-0.94)	0.71/0.86	0.80(0.67-0.94)	0.71/0.81
19kDa	A	0.60(0.49-0.72)	0.33/0.88	0.61(0.49-0.73)	0.33/0.90
19kDa	G	0.76(0.63-0.88)	0.86/0.67	0.74(0.58-0.90)	0.86/0.67

Receiver operator curve (ROC) analysis was used in determining the accuracy (sensitivity/specificity) of each serodiagnostic marker. ORD individuals comprised 21 LTBI and 21 QFT-ve ORD. Abbreviations: Ig = Immunoglobin; vs = versus; AUC = Area under the curve; CI = Confidence Interval.

### Evaluation of antibodies in discriminating between active TB disease and LTBI

**Figure 3:**
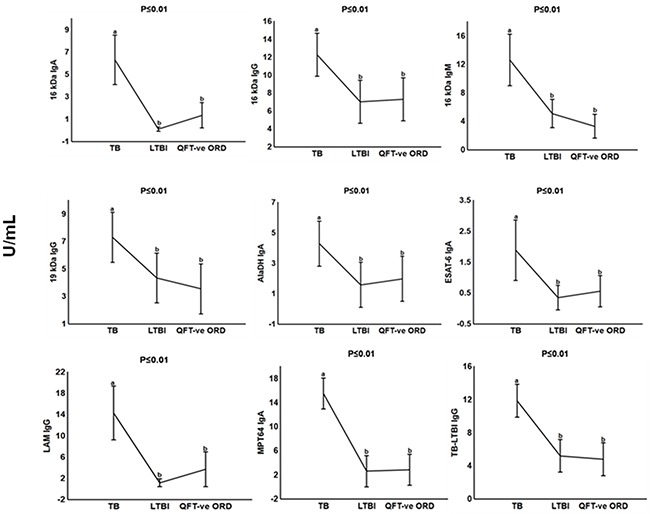
Plasma concentrations of serodiagnostic markers in individuals with tuberculosis, latently infected tuberculosis and QFT-negative other respiratory diseases Concentrations of plasma serodiagnostic markers were measured in 21 tuberculosis patients, 21 latently infected tuberculosis individuals and 21 QFT-negative other respiratory diseases. Representative plots are shown for diagnostic markers showing significant differences between groups and the vertical bars denote 95% confidence intervals. Significant difference between different groups p<0.05 is shown with the different alphabetical letters. The same alphabetical letters are used when there is no significant difference p>0.05 between the different groups. Corrections were applied within analyses of markers using Fischer Least Significant Differences post hoc testing or Games Howell post hoc depending on homogeneity of variance. All the reported significant p-values were adjusted.

**Figure 4:**
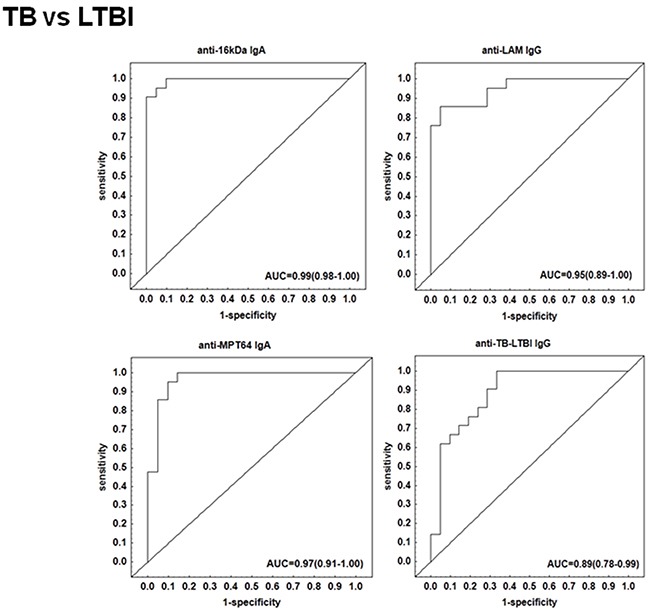
Receiving operating characteristics (ROC) curves of top single serodiagnostic markers for discriminating 21 active tuberculosis patients from 21 latently infected individuals

We performed a subgroup analysis and compared the antibody titres of the TB patients to those with other respiratory diseases who were QFT-positive. The IgA, IgG and IgM levels against 16 kDa, 19 kDa, AlaDH, ESAT-6, LAM, MPT64 and TB-LTBI protein antigens were significantly higher (p <0.01 in all cases) (Figure 3). When the discriminative abilities of the different antibodies were evaluated by ROC curve analysis, the four antibodies anti-LAM IgG, anti-TB-LTBI IgG, anti-MPT64 IgA, and anti-16 kDa IgA were the most accurate. The AUC for anti-16 kDa IgA was 0.99 (95% CI, 0.98-1.00), and that for anti- MPT64 IgA was 0.97 (95% CI, 0.91-1.00) (Figure 4). Both antibodies differentiated between active TB and LTBI with a sensitivity of 95% and specificity ≥90%, respectively (Table 2).

### Utility of multi-marker combinations to diagnose active TB

We evaluated the diagnostic abilities of combinations between the different antibodies in differentiating active TB from non-TB, or, alternatively from LTBI, using general discriminant analysis (GDA) models. For differentiating TB disease from other respiratory diseases, the most optimal biosignature was a three-marker serodiagnostic model comprising anti-TB-LTBI IgG, anti-Tpx IgG and anti-MPT64 IgA. This model correctly classified 95.2% (20 from 21) of the TB patients and 97.6% (40 from 41) of the ORD cases in the resubstitution classification matrix, with an overall accuracy of 96.8%, with the same level of accuracy after leave-one-out cross validation (Table 3). For discriminating between active TB and LTBI, the before-mentioned 3 markers plus anti-LAM IgA (a four-marker signature), classified both groups (TB disease or LTBI) with an accuracy of 100% in the resubstitution classification matrix, and an accuracy of 95.2% after leave-one-out cross validation (Table 3).

**Table 3:** Accuracies of seroantigen combinations to distinguish between TB and ORD, or LTBI, after general discriminant analysis

TB vs ORD					
Antigen combination	Resubstitution classification matrix			Leave-one-out cross-validation	
	%TB	% ORD	% Accuracy	% TB	% ORD
Anti-TB-LTBI IgG	95.23 (20/1)	97.61 (1/41)	96.8	95.23 (20/1)	97.61 (1/41)
Anti-Tpx IgG					
Anti-MPT64 IgA				PPV: 0.95 (95% CI; 0.74-0.99)	
				NPV: 0.97 (95% CI; 0.85-0.99)	

TB vs LTBI					
Antigen combination	Resubstitution classification matrix			Leave-one-out cross-validation	
	%TB	% LTBI	% Accuracy	% TB	% LTBI
Anti-LAM IgA	100.0 (21/0)	100.0 (0/21)	100.0	95.23 (20/1)	95.23 (0/21)
Anti-TB-LTBI IgG					
Anti-Tpx IgG					
Anti-MPT64 IgA				PPV: 0.95 (95% CI; 0.74-0.99)	
				NPV: 0.95 (95% CI; 0.75-0.99)	

The predictive abilities of the optimal combination of serodiagnostic markers to differentiate between active TB (n=21), definite LTBI (n=21) (IGRA^+^) or ORD individuals (IGRA^+^ and IGRA^-^ combined) (n=41) was investigated using best subsets general discriminant analysis and a leave-one-out cross-validation table was constructed using the variables that were included in the optimal classification model. PPV=Positive predictive value, NPV=Negative predictive value.

### Differential antibody responses in TB patients undergoing anti-TB treatment

**Figure 5:**
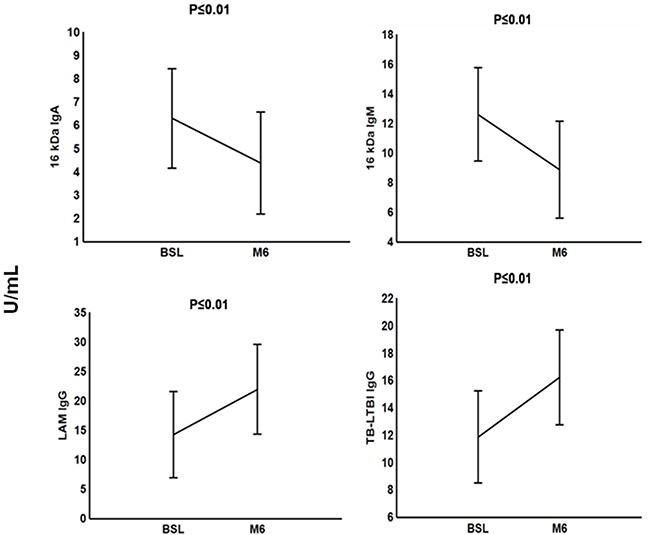
Plasma concentrations of serodiagnostic markers of tuberculosis patients during anti-TB treatment Concentrations of plasma serodiagnostic markers were measured in 21 TB patients at baseline (BSL) and in 19 TB patients who were followed up at end of month 6 anti-TB treatment. Representative plots are shown for diagnostic markers showing significant differences between the two time points and the vertical bars denote 95% confidence intervals. Corrections were applied within analyses of markers using Fischer Least Significant Differences post hoc testing or Games Howell post hoc depending on homogeneity of variance. All the reported significant p-values were adjusted.

Next, we investigated whether antibodies reflect the response to TB treatment. This analysis was only done in the 19 out of the 21 active TB patients for whom samples were available at the end of anti-TB treatment because 2 of the patients with TB disease were lost to follow up. We found significant low pre-treatment IgG responses to LAM and the TB-LTBI antigen combination compared to M6 TB treatment. However, we observed decreased significant levels of anti-16 kDa IgA and anti-16 kDa IgM after successful anti-TB treatment at M6 (Figure 5).

## DISCUSSION

The WHO does not support currently available commercial serologic tests for TB diagnosis but rather encourages further research to develop new tests with improved performance [3]. Performance targets for point-of-care tests in adults with smear-positive pulmonary TB include a sensitivity and specificity of 95%, while sensitivity in smear-negative cases should attain 60-80% and specificity of 95%. In extrapulmonary TB, a sensitivity of 80% and specificity of 90% have been proposed [16]. The results of this current study suggest that IgA and IgG responses against highly purified known and also relatively novel mycobacterial antigens, are promising due to the accuracies of individual and combination of marker above 95%. However, this is a small pilot study that requires further validation.

We have previously tested similar antibodies (anti-LAM IgG, anti-Tpx IgA). Results from the study showed that anti-LAM IgG was the best single marker discriminating TB from non-TB subjects and was also included in a five-marker combination with an improved performance. However, at that time, most of the controls were household contacts of active TB cases [6].

Previous studies have also found increased anti-TB antigen antibody levels in LTBI participants [4, 5]. In contrast, in this study, we found that the inclusion of the LTBI group did not affect test performance. Future research should investigate, whether recent household contacts of active TB cases affects serology compared to non-household contacts with LTBI. It is unknown whether increased anti-TB antigen antibody levels characterize a subgroup of LTBI individuals with high risk for progression to active TB, whose identification via antibodies would urgently be desirable [4, 17–19].

Much emphasis has been placed on the evaluation of IgG in most serological studies [5, 20, 21] with less attention directed towards IgA or IgM [22, 23]. IgA is produced particularly at mucosal sites. Like IgG, both mucosal and systemic IgA have protective effects and can trigger pro-inflammatory response [24, 25]. And IgM is associated with acute infection. For these reasons, the evaluation of IgA and IgM antibodies, besides IgG, makes sense. In the present study, IgA were in part more accurate than their IgG counterparts. In a previous study by Legesse *et al*., [26] IgA responses to protein antigens (ESAT-6/CFP-10 and Rv2031) were also found to be more accurate for diagnosis of active TB than IgG responses in TB endemic settings.

The immunodiagnostic potential of several antigens has been reported [6, 27–29]. Out of all the antigens tested, LAM, the two proteins 16kDa and MPT64, and the antigen combination TB-LTBI best discriminated active TB from the other two groups with high sensitivity and specificity. The 16 kDa polypeptide is a member of the low molecular weight α-crystallin heat shock proteins. It is a dominant protein produced under oxygen starvation or passive growth phase and also important for the replication of bacteria in macrophages [30, 31]. In the present study, 16 kDa elicited IgA, IgG and IgM responses in the plasma of the TB patients. Similarly, measuring 16 kDa antigen against all three isotypes, Raja and colleagues [32] found a combined specificity of 93% for serological detection in sputum and culture confirmed pulmonary TB patients. LAM is a component of the *M. tuberculosis* cell wall [33] and it is a well researched *M. tuberculosis* antigen for TB diagnosis in serological studies [6, 34, 35]. LAM elicited pronounced IgG responses in TB patients in this study and this shows its potential in TB serodiagnostic even in endemic settings. Similarly, the serodiagnosis of active TB patients through LAM achieved a high degree of specificity [34]. In contrast to our previous study, IgG responses were only elicited by LAM in LTBI subjects compared to healthy controls. Antibodies against MPT64 a highly specific protein that is secreted by *M. tb, M. bovis and M. africanum* [36–38] could be specific in the detection of TB especially as its expression is less in BCG vaccines [39]. An IgA response to MPT64 discriminated TB from QFT negative non-cases and LTBI with sensitivity of 95%. An MPT64 antibody aptamer, showed serological potential in the diagnosis of pulmonary TB in sputum smear positive as well as sputum smear negative patients [40].

The combination of IgA and IgG responses may help to increase accuracy of serodiagnostic tests for active TB disease in TB endemic settings, as previously suggested [6]. Accuracies of combinations of antibodies revealed that the combined IgG responses to Tpx and TB-LTBI and IgA response to MPT64 best discriminated active TB from ORD with a positive predictive value of 0.95 (95% CI; 0.74-0.99) and negative predictive value of 0.97 (95% CI; 0.85-0.99). The same antibody combinations with the inclusion of anti-LAM IgA were found to give the best discrimination between active TB and LTBI with positive and negative predictive values of 0.95(95% CI; 0.74-0.99) and 0.95(95% CI; 0.75-0.99) respectively. The multivariate analysis in our previous study with mainly TB household contacts [6] also revealed that both Tpx and LAM antigens featured prominently in discriminating TB from non-TB with an accuracy of 86.2%. Also the serologic responses to these two consistent antigens should be further investigated in a well-designed cohort study. It has been suggested that using multi-antigen cocktails will increase the sensitivity of TB serodiagnosis above that of single antigens [41, 42]. This might be due to the differential expression of certain antigens during the stages of TB development [43, 44]. Of note, we mathematically found that also markers like anti-Tpx IgG and/or anti-LAM IgA, which performed less well in Univariate analysis (Table 2), were valuable contributors in the Multivariate analysis (Table 3), as they complemented very well other well performing biomarkers, such as anti-PT-64 IgA and TB-LTBI IgG (Table 3). In a way, anti-Tpx IgG and/or anti-LAM IgA are able to detect other active TB cases than e.g. anti-MPT-64 IgA, a biomarker, which already had an accuracy >90% on its own. Additionally, as pointed out, an important point may be the combination of separately measured specific IgG and IgA antibodies.

We found a significant decrease in the levels of anti-16 kDa IgM and anti-16 kDa IgA after a successful completion of anti-TB treatment. Imaz and Zerbini [45] also reported decreased levels of antibodies, however, only three years after the start of anti-TB chemotherapy. A study on humoral response of TB patients undergoing anti-TB treatment indicated that antibody levels against other antigens had no association with anti-TB chemotherapy [46].

## CONCLUSION

In summary, this study has shown that IgG and IgA antibody responses against single and multiple-antigen cocktails as well as multi-marker serologic models differentiated active cases from non-cases amongst people presenting with presumed TB regardless of LTBI status. Furthermore, our results suggest that the antibodies against the specific *M. tuberculosis* antigens tested in this study may be more useful for TB diagnosis than for monitoring treatment response. This result may encourage additional future efforts to investigate serologic responses in TB diagnostics research as such tests would be amenable to the development of rapid lateral flow-based test formats with application in field settings in a laboratory-free manner. However, future large scale prospective studies to include immunocompromised HIV co-infected patients are needed to further evaluate the validity of these results.

## MATERIALS AND METHODS

### Study population

Participants included in the present study were individuals presenting with signs and symptoms requiring investigation for TB, and were recruited as part of the recently concluded EDCTP-funded African European Tuberculosis Consortium (AE-TBC) study [12]. All study participants were recruited from a peripheral level health care centre, Fisantekraal, situated in the outskirts of Cape Town, South Africa. All participants presented with persistent cough lasting for more than 2 weeks and one of the following: fever, recent loss of weight, night sweats, haemolysis, chest pain or loss of appetite. The eligibility criteria for the study included age of between 18 and 65 years, and willingness to give written informed consent including for HIV testing. The exclusion criteria included severe anaemia (HB<10g/l), current anti-TB treatment, anti-TB treatment in the last 90 days, or taking quinolone or aminoglycoside antibiotics in the past 60 days, and not being resident in the study area for more than 3 months at presentation. Sputum samples were collected from all study participants and cultured using the MGIT method (BD Biosciences). Confirmation of the isolation of organisms of the *M. tuberculosis* complex in all positive cultures was carried by the Capilia TB test (TAUNS, Numazu, Japan). Additionally, 3 ml of blood were collected from the participants for the performance of Quantiferon-in Tube (QFT-IT) assay (Qiagen), which was carried out according to the manufacturer's instruction as previously described [13]. For the current study, we included 21 patients with culture positive TB and 42 with other lung diseases of which 21 had LTBI as defined by a positive QFT test and of which 21 were QFT-negative. These participants were randomly selected from Stellenbosch set of samples from the main study and according to availability of baseline and month 6 samples. All active TB patients received standard TB treatment according to South African National Tuberculosis Program and samples were collected from 19 of the TB participants at the end of TB treatment at month 6. This means, that out of the original 21 TB patients, samples were only collected from 19 of the participants at the end of month 6 TB treatment as two participants were lost due to follow up. None of the non-TB patients (LTBI and the QFT-negative) received anti-TB treatment. Ethical approval for the study was obtained from the Health Research Ethics Committee of the University of Stellenbosch (reference number N10/08/274) and written informed consent was obtained from each participant before the study.

### Sample collection and preparation

At enrolment, 10ml of whole blood were collected from all study participants directly into heparinized BD vacutainer tubes (BD Biosciences), and transported at ambient conditions within two hours of collection to the laboratory. The tubes were then centrifuged at 1200xg for 10 minutes, and plasma harvested and stored at -80°C until further use. Sample collection was repeated for 19 TB patients at months 6 following anti-TB treatment.

### Antigen preparation

Seven cloned and purified recombinant proteins of *M. tuberculosis* (Table 4) were used in the present study. For MPT64 expression, recombinant *M. smegmatis* mc^2^ 155 cells containing the MPT64 expression plasmid (with C-terminal histidine tag) were grown in animal source-free medium at 37°C overnight. The recombinant MPT64 was secreted into the culture medium by constitutive expression. The medium was harvested by centrifugation followed by buffer exchange on a Sephadex G25 column (GE Healthcare) into 10 mM NH_4_HCO_3_ buffer. The protein containing solution was applied onto Ni-NTA Superflow resin (Qiagen). MPT64 was eluted in a linear imidazole gradient. Highly pure MPT64-containing fractions were pooled and underwent a final buffer exchange on a Sephadex G25 column (GE Healthcare) into 10 mM NH4HCO3, pH 8,0. Aliquots of the protein solution were freeze-dried and stored below -20 °C. The production of the remaining protein antigens has been described previously: 19 kDa [4], AlaDH [14], ESAT-6 [11], 16 kDa [6], PstS1 [15], and Tpx [11]. We also evaluated two multiple antigen cocktails in this study. The first multiple antigen cocktail TUB contains PstS1, 16kDa and APA while the second TB-LTBI is composed of Tpx and L16. All recombinant proteins of *M. tuberculosis* antigens were prepared by LIONEX Diagnostics and Therapeutics, Braunschweig, Germany. A sample of highly purified *M. tuberculosis* LAM was kindly provided by Dr. Arend Kolk, Amsterdam.

**Table 4:** Recombinant antigens of *M. tuberculosis* used in this study

Antigens of *M. tuberculosis*	Rv no.	Mol.mass (kDa)	Reference(s)	Ig class
19 kDa glycolipoprotein, LpqH	Rv3763	16	[4]	IgA, IgG
AlaDH	Rv2780	38.7	[14]	IgA, IgG
ESAT-6	Rv3875	9.9	[11]	IgA, IgG
HSP16.3, HSPX, 14 kDa, 16 kDa, ACR	Rv2031c	16.3	[6]	IgA, IgG, IgM
LAM	–	–		IgA, IgG
MPT64	Rv1980c	24.8	[47]	IgA, IgG
PstS1, 38 kDa	Rv0934	38.2	[15]	IgG
Tpx, CFP20	Rv1932	16.9	[11]	IgA, IgG

### Enzyme-linked immunosorbent assay

All pre-coated IgG, IgA and IgM ELISA test kits and reagents against *M. tuberculosis* antigens were provided by LIONEX Diagnostics and Therapeutics, Braunschweig, Germany. The actual ELISA measurements of the human plasma samples were performed in South Africa, including the evaluation of the results. In brief, human plasma was diluted 1:200 in PBS pH 7.5/0.05% BSA buffer. One hundred μl of the diluted plasma and ready-to-use standards were pipetted into the antigen-coated wells of the microtiter plate in duplicates. After 60 minutes incubation while shaking (45 minutes incubation for Immunoglobulin (Ig) M) at 37°C, the well contents were emptied and plates washed three times with 300 μl/well PBS-T (0.15 M PBS, pH 7.5/0.05% Tween-20). 100 μl/well of ready to use anti-human-IgG-conjugate (diluted 1:40 000), anti-human-IgA (1:12 000) or anti-human-IgM (1:12 000) antibodies were added to the wells and the plates incubated for 30 minutes at 37°C while shaking. After a second washing step, the enzyme activity was assayed by rapidly adding 100 μl/well of substrate tetramethylbenzidine (TMB) with a further incubation for 20 minutes at 37°C in the dark. The colour development was ended by the rapid addition of 100 μl/well 0.2 M H_2_SO_4_ stop solution. The absorbance was measured at 450 nm (OD_450_) with a 620 nm (OD_620_) reference filter using an automatic microplate reader (iMark™ Microplate absorbance reader, BIO RAD, USA). The mean OD of the blank wells was subtracted from the sample values.

### Statistical analysis

For the evaluation of the diagnostic potential of serodiagnostic markers, statistical differences in the concentrations of markers between active TB and individuals with other respiratory diseases were analysed by analysis of variance (ANOVA) with Fisher Least Significant Difference (LSD) *post hoc* test or Mann-Whitney U test depending on the normality of the distribution. In cases where Levene's test rejected the assumption of homogeneity of variance, weighted means were reported and the Games-Howell post-hoc test conducted. Receiver operating characteristics (ROC) curve analysis was used in evaluating the accuracy of the different markers. To investigate the predictive abilities for the optimal combination of serodiagnostic markers for differentiating TB disease and individuals with ORD, general discriminant analysis (GDA) was performed. The best subsets method was employed in determining optimal subsets of variables that gave the best prediction. Using the best variables that were included in the optimal classification model, a leave-one-out cross validation table was constructed. For the investigation of markers that could be useful in monitoring of the response to TB treatment, the change in the concentrations of markers during TB treatment was analyzed using mixed model repeated measures analysis of variance (ANOVA) with Fisher Least Significant Difference (LSD) post hoc test. A level of 5% significance was used as a guideline for the determination of significance associations. All statistical analysis except ROC analysis was performed using Statistica software (Statsoft, Ohio, USA). ROC analysis was done using R program language.
